# Telemedicine for Children With Cerebral Palsy Before, During, and After the COVID-19 Pandemic: An Australian Cohort Study

**DOI:** 10.1177/08830738251339960

**Published:** 2025-05-21

**Authors:** Simon P. Paget, Sarah McIntyre, Amy von Huben, Kirsty Stewart, Tracey Williams, Emma Maly, Katrina Ford, Sue Woolfenden, Natasha Nassar

**Affiliations:** 1 The Children's Hospital Westmead Clinical School, 522555 Faculty of Medicine and Health, University of Sydney, Sydney, New South Wales, Australia; 2 8538 Kids Rehab, The Children's Hospital at Westmead, Westmead, New South Wales, Australia; 3 Cerebral Palsy Research Institute, 522555 Faculty of Medicine and Health, University of Sydney, Sydney, New South Wales, Australia; 4 226915 Menzies Centre for Health Policy & Economics, Faculty of Medicine and Health, University of Sydney, Sydney, New South Wales, Australia; 5 CP Quest, 383637 Cerebral Palsy Research Institute, 522555 Faculty of Medicine and Health, University of Sydney, Sydney, New South Wales, Australia; 6 Community Paediatrics Research Group, Central Clinical School, 522555 Faculty of Medicine and Health, University of Sydney, Sydney, New South Wales, Australia

**Keywords:** COVID-19, cerebral palsy, telemedicine

## Abstract

**Objective:** To examine telemedicine use in children with cerebral palsy before, during and since the COVID-19 pandemic. **Methods:** A retrospective cohort study of 1162 children with cerebral palsy (40.3% female, birth years 2005-2017), attending specialist outpatient clinics at 2 pediatric hospitals in New South Wales, Australia. We categorized outpatient visits from January 2018 to May 2023 as in-person or telemedicine and compared usage pre–, during, and post–COVID-19 periods. Neighborhood socioeconomic disadvantage and geographical remoteness were defined by residential postcode. **Results:** Of 48 896 outpatient encounters, 11 929 (24.4%) used telemedicine. Telemedicine rates increased during COVID-19 (20.2 per 100 persons/month) and declined post–COVID-19 (15.2 per 100 persons/month, *P* < .001). Neighborhood socioeconomic disadvantage was associated with higher median outpatient and telemedicine encounter rates. Regional/remote children had lower median outpatient and telemedicine rates. **Conclusion:** Telemedicine use declined since lifting of COVID-19 pandemic restrictions. Further support will be required to sustain rates and learn from pandemic experiences.

Telemedicine describes the delivery of health care services, by health care professionals using information and communications technologies for the exchange of information.^1^ This is particularly pertinent when distance is a critical factor. Telemedicine includes outpatient encounters where consultation occurs through video or telephone, but the term is often used to include broader approaches including the sharing of medical images and remote monitoring of vital signs (eg, heart rate, blood pressure, temperature).^2^ Digital technologies, such as telemedicine, have been identified as a key mechanism of improving access to health care.^3^ Studies have shown that telemedicine can improve equity of access to some health care services,^4,5^ particularly for patients living in regional and remote areas.^6,7^

Cerebral palsy is a common childhood chronic health condition, and the most common cause of physical disability with a birth prevalence of 1.5 per 1000 live births in Australia.^8^ Children with cerebral palsy are frequent users of health care^9^: one quarter use outpatient services more than once monthly, and across multiple specialties,^10^ and one-third live outside metropolitan areas.^8^ Complexities of travel and managing time away from work and other caring roles may make in-person attendance for health care consultations challenging. Telemedicine, therefore, may have an important role in improving access to health care and health outcomes for these children.

The COVID-19 pandemic was associated with a rapid and substantial increase in telemedicine as public health restrictions and the direct impacts of the virus limited access to in-person health care,^11^ including for children with cerebral palsy.^12^ Studies conducted during this period, particularly among children with cerebral palsy and other neurodevelopmental disorders, described increased telemedicine use for medical consultations^12^ and explored the potential for telemedicine, for example, to provide rehabilitation programs to improve gross motor function^13^ or manage common comorbidities such as epilepsy.^14^ One study suggested that telemedicine did not suit all aspects of care for children with neurodevelopmental disabilities.^15^ Others studies highlighted the “digital divide”^16^: inequities in telemedicine access^14^ that are also seen more broadly in society,^17^ such as availability of information technologies (eg, smartphones, broadband Internet) and usability of digital platforms for those with less experience of technology.^18^ We aimed to examine patterns of telemedicine use for specialist outpatient clinics in children with cerebral palsy in time periods before, during, and since the COVID-19 pandemic. We also aimed to compare telemedicine use in children with cerebral palsy with different levels of neighborhood socioeconomic disadvantage and/or residential geographical remoteness.

## Patients and Methods

We conducted a retrospective cohort study using administrative health data. Ethical approval was granted by Sydney Children's Hospitals Network Human Research Ethics Committee (2019/ETH11829). We identified children with cerebral palsy, born from 2005 to 2017, from the New South Wales / Australian Capital Territory CP Register (n = 1764). The New South Wales / Australian Capital Territory CP Register is a population-level database with multiple ascertainment strategies.

We identified outpatient encounters for these children at 2 tertiary government pediatric hospitals in metropolitan Sydney, New South Wales, Australia (Sydney Children's Hospital, Randwick and the Children's Hospital at Westmead). Combined, these 2 hospitals provide the majority of specialty pediatric services for children living in New South Wales. Data were obtained from the hospitals’ nonadmitted patient data collection, which collects patient-level activity for all clinical and/or therapeutic services provided in nonadmitted settings at the hospitals for reporting purposes. The health care for nonadmitted patients captured in the nonadmitted patient data collection is provided under a government universally funded system (either state or federally funded) and without a fee to the patient.

Data available from the New South Wales / Australian Capital Territory CP Register included age, sex, clinical information (Gross Motor Function Classification System, epilepsy, intellectual disability). Postcode of residence was determined from nonadmitted patient data, or where not available, from the New South Wales / Australian Capital Territory CP Register. Neighborhood socioeconomic disadvantage was determined by assigning each individual residential postcode to its corresponding Australian Bureau of Statistics Index of Relative Socioeconomic Disadvantage score.^19^ The Index of Relative Socioeconomic Disadvantage score is calculated using collective socioeconomic characteristics (eg, income, employment status, education, transport) of the people living in a particular area. The Index of Relative Socioeconomic Disadvantage was categorized into quintiles (quintile 1 being the most disadvantaged and quintile 5 being the least disadvantaged) for most analyses; Index of Relative Socioeconomic Disadvantage quintile 1 was also split into 2 categories (deciles 1 and 2) to look more closely for evidence of digital divide. Geographical remoteness was defined using the Australian Statistical Geography Standard, which categorize each residential postcode as a major city, inner/outer regional, and remote/very remote area) based on ease of access to services via road network.^20^

Data from the nonadmitted patient records were available from January 1, 2018, to May 31, 2023. We defined 3 time periods: a pre–COVID-19 time period (January 1, 2018, to February 29, 2020 [26 months]), a COVID-19 time period (March 1, 2020, to December 31, 2021 [22 months]), and a post–COVID-19 period (January 1, 2022, to May 31, 2023 [17 months]). The COVID-19 time period corresponded to the commencement of COVID-19–related restrictions in Australia (March 2020) to the easing of restrictions (eg, opening of international borders) in December 2021, and included periods of stringent public health measures in New South Wales during March-July 2020 and July-October 2021. We categorized the modality of outpatient encounters as in-person or telemedicine (including telephone and audiovisual encounters). We excluded encounters with no direct patient contact (eg, case conferences, case planning, email). We identified the 4 most frequent clinical specialty types (tier 2 class) based on frequency of (either in-person or telemedicine) encounters during the study period.

**Figure 1.**
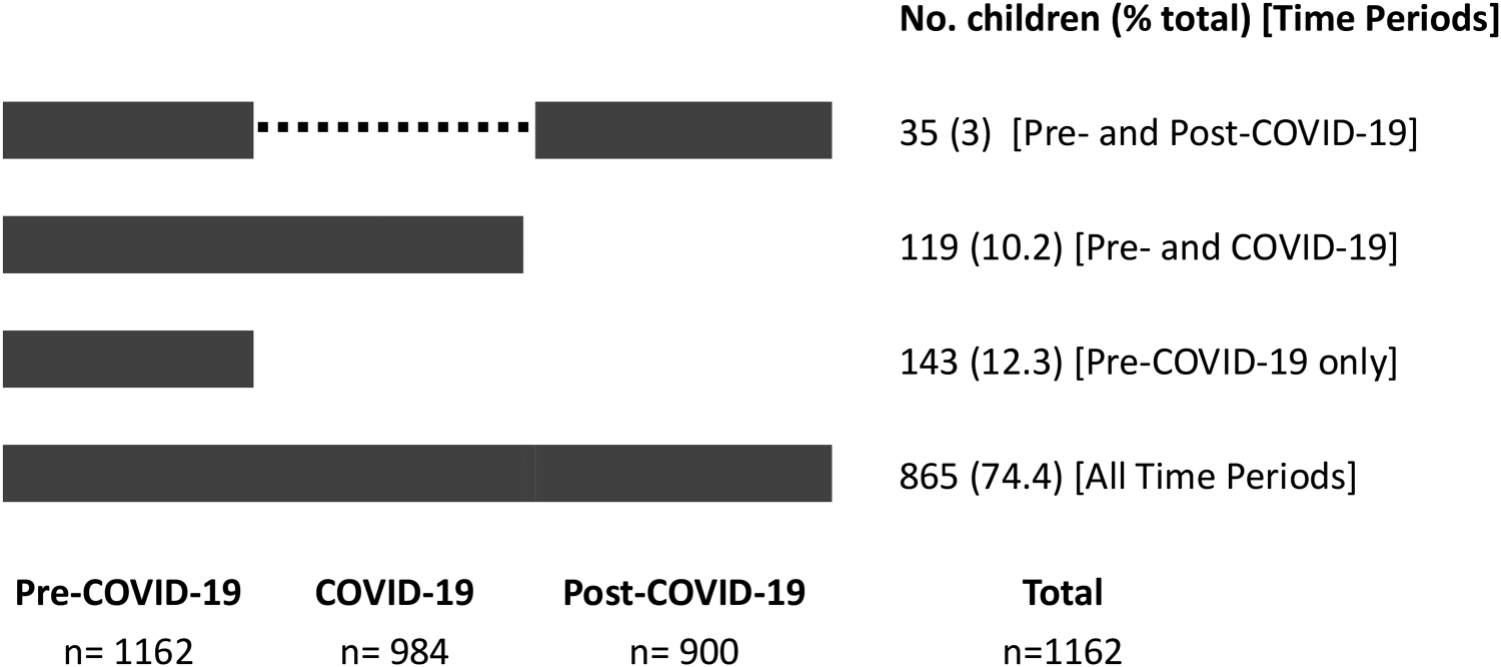
Frequency of children with cerebral palsy having one or more outpatient encounters at 2 children's hospitals in time periods during the COVID-19 pandemic.

In order to improve understanding of how telemedicine use changed during the study, we included all children in analyses who had at least 1 outpatient encounter in the pre–COVID-19 time period (Figure 1). We undertook descriptive analyses of the children and outpatient encounters. We determined the representativeness of our study population by comparison to children in the New South Wales / Australian Capital Territory CP Register using contingency tables, *t* tests for continuous variables (age), χ^2^ tests for categorical variables, and Fisher exact test where expected frequencies were <5.

We performed descriptive analyses of total outpatient encounters and telemedicine encounters, including for the 4 most frequent clinical specialty types. We determined average monthly rates of outpatient encounters (total, telemedicine) per 100 persons reported as medians and interquartile ranges (IQRs), comparing rates between demographic and clinical descriptor groups (eg, sex, socioeconomic disadvantage, geographical remoteness, Gross Motor Function Classification System, epilepsy, intellectual disability).

To determine changes in telemedicine over time, we determined monthly rates of total outpatient encounters and telemedicine encounters (median encounters per 100 persons per month) and the proportion of total outpatient encounters by telemedicine. We compared median rates across the 3 time periods using the Kruskal-Wallis test. We compared changes in rates across time periods by age groups. Initial analyses suggested significant variation in rates of total outpatient encounters by calendar month (lower in January and December, corresponding to a “low activity” period including Christmas and summer holidays in New South Wales) (Supplementary Figure S1). We therefore performed sensitivity analyses calculating rates of total outpatient encounters and telemedicine encounters for each time period removing encounters in January or December. To further examine changes in telemedicine over time we analyzed the monthly proportion of total outpatient encounters provided by telemedicine using joinpoint regression. Joinpoint is a trend analysis software that describes changes in data by connecting several different line segments on a log scale at “joinpoints.” Analysis starts with a minimum number of joinpoints (ie, zero joinpoints), and adds joinpoints to the model where they improve model fit, using Monte Carlo permutation to test significance.^21^ The joinpoint regression also calculates monthly percentage change and 95% confidence intervals.

We performed stratified analyses of rates of total outpatient encounters, telemedicine encounters, and proportion of telemedicine between groups of children based on socioeconomic disadvantage (Index of Relative Socioeconomic Disadvantage 1 vs 2-5) and geographical remoteness (major cities vs regional and remote) using the Kruskal-Wallis test. Analyses were performed in SAS 9.4 (SAS Institute, Cary, NC) and Joinpoint Regression Program, V5.0.2 (National Cancer Institute, Bethesda, MD). The report was completed consistent with Strengthening the Reporting of Observational Studies in Epidemiology (STROBE) statement guidelines.^22^

This study was conducted in partnership with 2 research partners (EM, KF) with lived experience from CP Quest (https://cerebralpalsy.org.au/get-involved-research/cp-quest/). Discussions with both partners guided the prioritization of research questions and supported contextual understanding of the data and initial results. These insights were used to inform the discussion of this article.

## Results

We identified 1162 children with cerebral palsy (59.7% male, mean age at study commencement 7.3 years, standard deviation 3.4 years) who had 1 or more outpatient encounters in the pre–COVID-19 and in the COVID-19 and/or post–COVID-19 time periods (Table 1). Almost three-quarters of children (74.4%) had outpatient encounters in all 3 time periods (Figure 1), and more than 85% (n = 993) had 1 or more telemedicine encounter. Among children with outpatient encounters only in the pre–COVID-19 time period (n = 144), 26.6% (n = 38) had only 1 encounter in total. Children with outpatient encounters only in the pre–COVID-19 time period were broadly similar to those with encounters in all 3 periods (differences noted in proportions of Gross Motor Function Classification System I-III (73.4% vs 68.9%) (*P* < .0001), epilepsy (26.6% vs 30.4%) (*P* = .01), and intellectual disability (40.6% vs 49.5%) (*P* = .04). Compared with children identified in the New South Wales / Australian Capital Territory CP Register without an outpatient encounter, children with at least 1 encounter were younger, more likely to live in major cities and neighborhoods with less socioeconomic disadvantage, and more likely to have severe cerebral palsy (Gross Motor Function Classification System IV-V) and/or comorbidities**.**

**Table 1.** Demographic and Clinical Details of Children With Cerebral Palsy Attending Outpatient Encounters at 2 Children's Hospitals, Rates of Total Outpatient Encounters, and Telemedicine Encounters.

Demographic/clinical factor	Total children attending outpatient services, n (%)	Total encounters per 100 persons, median (IQR)	*P* value	Telemedicine encounters per 100 persons, median (IQR)	*P* value
Total	1162 (100)	66.4 (56.5-73.5)		14.3 (11.9-18.2)	
Sex			.71		.83
Male	694 (59.7)	67.3 (57.5-73.1)		14 (11.5-18.9)	
Female	468 (40.3)	65.2 (53.2-76.7)			
Socioeconomic disadvantage (quintiles)			<.001		<.001
1 (most disadvantaged)	208 (18.2)	77.9 (65.4-89.9)		16.3 (13.5-21.2)	
2	157 (13.7)	49 (35.7-55.4)		13.4 (9.6-15.9)	
3	248 (21.7)	55.2 (48-73)		13.7 (10.9-16.9)	
4	237 (20.7)	65.4 (56.5-77.6)		15.2 (10.5-19.8)	
5 (least disadvantaged)	294 (25.7)	68 (57.1-77.9)		14.6 (11.9-20.4)	
Geographical remoteness			<.001		<.001
Major city	810 (70.7)	73.2 (59.9-80.6)		15.1 (12.6-19.4)	
Inner regional	302 (26.4)	49.3 (42.4-60.6)		13.2 (9.6-15.6)	
Outer regional / remote	34 (3.0)	41.2 (26.5-61.8)		11.8 (8.8-17.6)	
GMFCS			<.001		<.001
I-III	816 (70.2)	47.3 (39.5-53.3)		9.7 (8-12.4)	
IV-V	303 (26.1)	116.5 (102.9-127.3)		27.1 (22.4-35)	
Unknown	43 (3.7)	55.8 (39.5-72.1)		11.6 (9.3-18.6)	
Epilepsy			<.001		<.001
Yes	338 (29.1)	85.8 (68-93.3)		19.5 (16.2-25.1)	
None or resolved	665 (57.2)	51.7 (43-58.6)		10.7 (8.4-13.4)	
Unknown	159 (13.7)	55.8 (39.5-72.1)		11.6 (9.3-18.6)	
Intellectual disability			<.001		<.001
Yes	550 (47.3)	85.8 (68-93.3)		19.5 (16.2-25.1)	
None	432 (37.2)	42.4 (35.2-49.1)		8.1 (6.7-10.9)	
Unknown	180 (15.5)	60 (47.2-70)		12.8 (10-18.9)	

Abbreviations: GMFCS, Gross Motor Function Classification System; IQR, interquartile range.

A total of 48 896 encounters were recorded between January 2018 and May 2023, one-quarter (n = 11 929, 24.4%) involved telemedicine. In the pre–COVID-19 time period, only 1.4% (n = 51) telemedicine encounters used an audiovisual medium (the remainder using telephone). The proportion of audiovisual telemedicine encounters increased during the COVID-19 period (29.1%, n = 1590) and was sustained post–COVID-19 (19%, n = 525). Rates of total encounters and telemedicine encounters were higher in children in Index of Relative Socioeconomic Disadvantage quintile 1 (most disadvantaged), in children living in major cities, and in children with Gross Motor Function Classification System IV-V (compared with those Gross Motor Function Classification System I-III), and in those with epilepsy and/or intellectual disability (Table 1).

**Figure 2.**
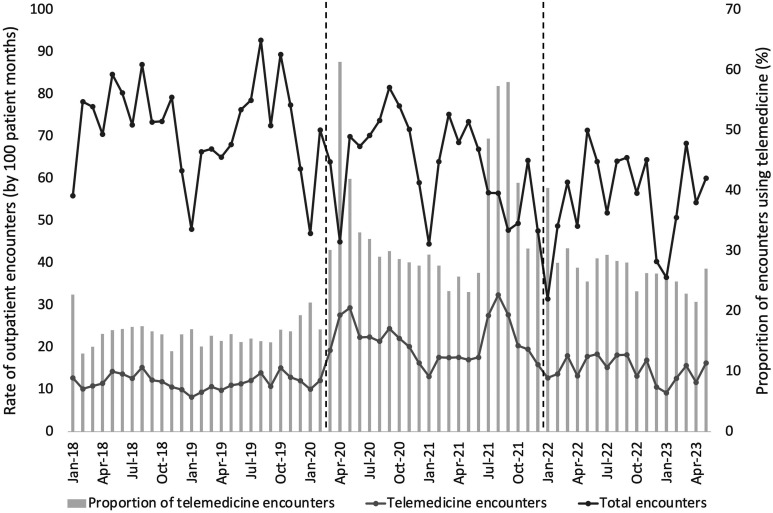
Rates of total and telemedicine outpatient encounters and proportion of telemedicine encounters for children with cerebral palsy at 2 specialist children's hospitals. Dashed lines indicate change between COVID-19 time periods.

Monthly rates and proportion of total and telemedicine encounters changed over time (Figure 2). The median rate of total encounters decreased in consecutive time periods from 76.1 per 100 persons per month (interquartile range [IQR] 66.4-78.6; pre–COVID-19) to 65.6 (IQR 56.5-71.7; COVID-19) and 56.5 (IQR 48.8-64.1; post–COVID-19), *P* < .001). This decline was also noted across consecutive years when adjusting for age group (Supplementary Table 1). The median rate of telemedicine encounters increased between the pre–COVID-19 (rate 11.7 per 100 persons per month [IQR 10.6-12.7]) and COVID-19 time periods (rate 20.2 per 100 persons per month [IQR 17.6-24.4]) and decreased in the post–COVID-19 time period (rate 15.2 per 100 persons per month [IQR 12.7-17.8]) (*P* < .001). Sensitivity analyses (excluding December and January) showed similar relationships between time periods. Differences in patterns of telemedicine rates and proportions were identified between major specialty types (Table 2). Some specialties (Rehabilitation and Neurology) provided a higher proportion of outpatient encounters using telemedicine across time periods (eg, 29.9% and 39.6% in the pre–COVID-19 period, respectively), whereas others (Allied Health, Orthopaedics) provided a lower proportion (eg, 4.7% and 3.4% in the pre–COVID-19 period, respectively). The proportion of encounters provided using telemedicine increased in all specialty groups during the COVID-19 period and declined post–COVID-19. Post–COVID-19, higher telemedicine use was sustained in Rehabilitation (40.0%) and Neurology (61.8%) specialties.

**Table 2.** Proportion of Outpatient Encounters Provided Using Telemedicine for Children With Cerebral Palsy by Major Specialty Types During the COVID-19 Pandemic.

	Rehabilitation (n = 15 813)		Allied health (n = 150 105)		Neurology (n = 3120)		Orthopedics (n = 4862)		Other (n = 9923)	
	Telemedicine	In-person	Telemedicine	In-person	Telemedicine	In-person	Telemedicine	In-person	Telemedicine	In-person
Pre–COVID-19 n (%)	1938 (29.9)	4551 (70.1)	323 (4.7)	6601 (95.3)	559 (39.6)	854 (60.4)	61 (3.4)	1738 (96.6)	666 (12.8)	4519 (87.2)
COVID-19 n (%)	2768 (48.8)	2905 (51.2)	320 (6.5)	4578 (93.5)	824 (74.6)	280 (25.4)	242 (14.5)	1428 (85.5)	1303 (45.5)	1562 (54.5)
Post–COVID-19 n (%)	1459 (40)	2192 (60)	209 (6.4)	3074 (93.6)	418 (61.8)	258 (38.2)	103 (7.4)	1290 (92.6)	736 (39.3)	1137 (60.7)

**Figure 3.**
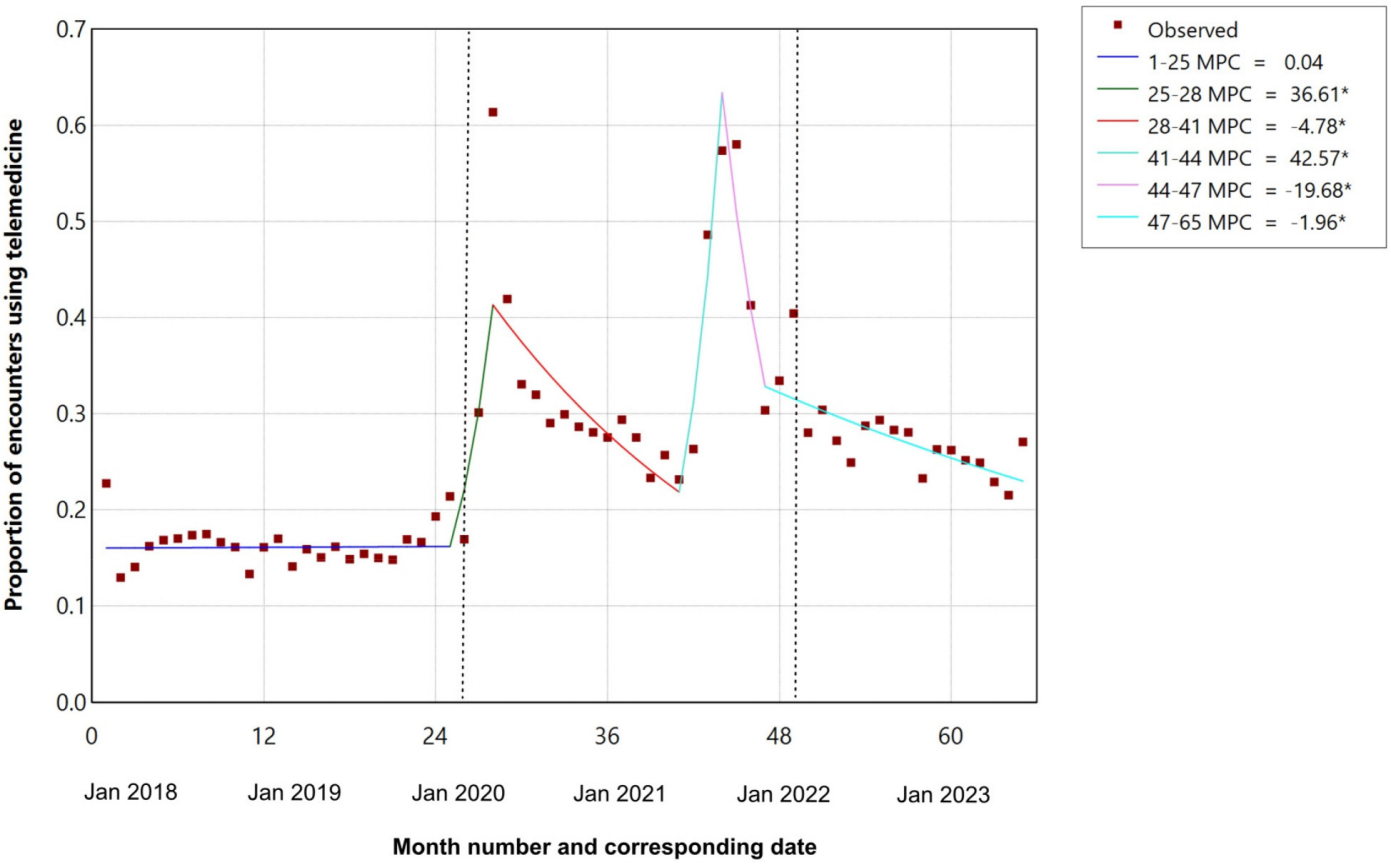
Joinpoint regression of proportion of outpatient encounters using telemedicine for children with cerebral palsy (January 2018 and May 2023).

There were notable peaks in telemedicine use during the COVID-19 pandemic in March 2020 (61.4% total encounters) and August and September 2021 (57.4% and 58.0% total encounters, respectively) (Figure 2). Joinpoint analysis of the proportion of monthly outpatient encounters provided by telemedicine is shown in Figure 3. In the pre–COVID-19 period the proportion of telemedicine was stable (16%). There were 2 periods of rapid increase in telemedicine starting in February 2020 (monthly percentage change 36.7%) and May to August 2021 (monthly percentage change 24.9%) separated by a 13-month period where telemedicine declined by 4.6% per month. After August 2021, the proportion of encounters provided using telemedicine rapidly decreased by 19.7% per month for 3 months and after November 2021 continued to decline more slowly by 2% per month.

*The monthly percentage change (MPC) is significantly different from zero at the alpha = 0.05. Dashed lines indicate change between COVID-19 time periods.

**Figure 4.**
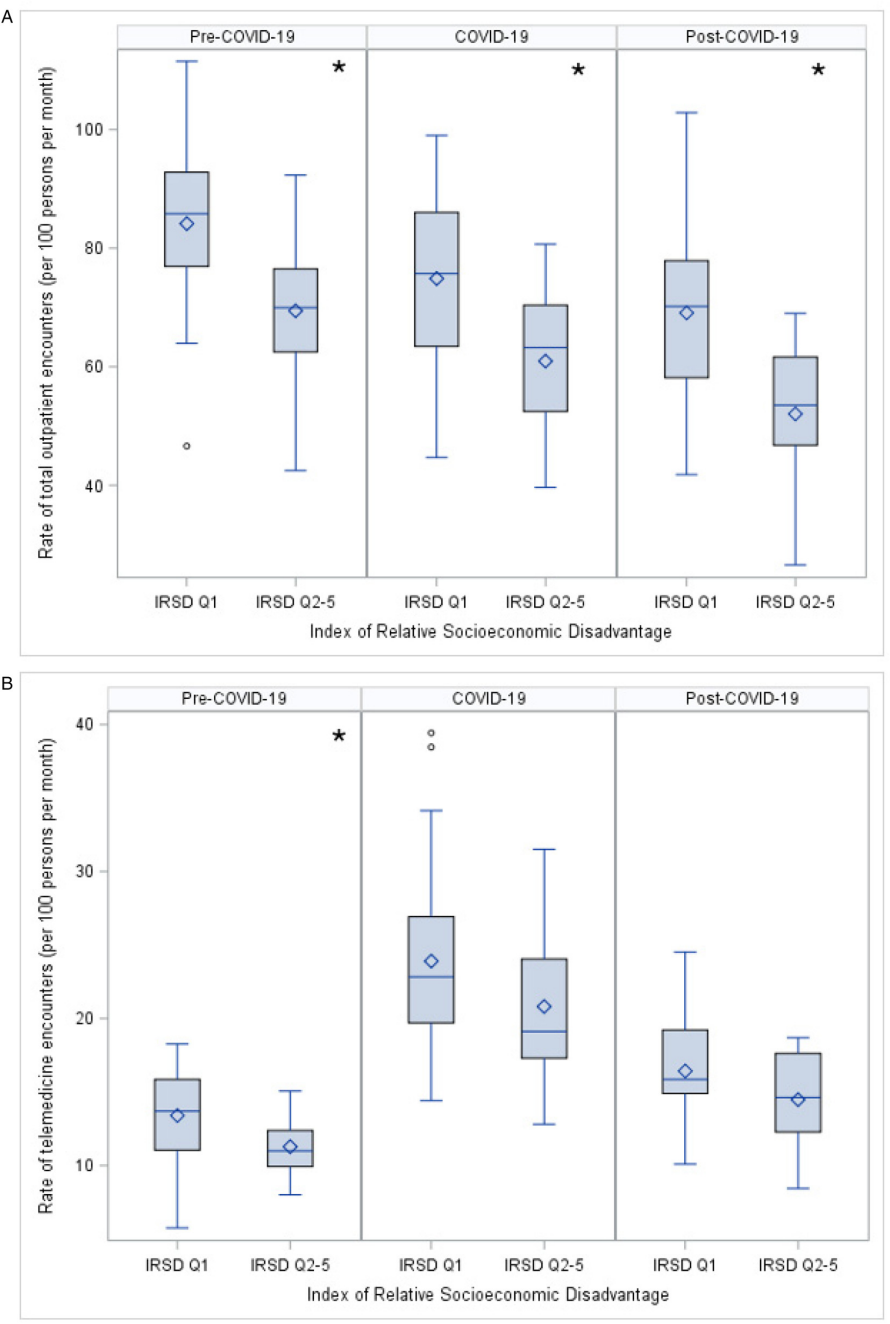
Comparison of (A) rate of outpatient encounters and (B) rate of telemedicine encounters for children with cerebral palsy living in most socioeconomically disadvantaged areas (IRSD quintile 1) and children with cerebral palsy living in less disadvantaged areas (IRSD quintiles 2-5).

Children living in neighborhoods of Index of Relative Socioeconomic Disadvantage quintile 1 (most disadvantaged) had a higher median rate of outpatient encounters than children living in neighborhoods of Index of Relative Socioeconomic Disadvantage quintiles 2-5 in all time periods (Figure 4A). Children living in Index of Relative Socioeconomic Disadvantage quintile 1 had a slightly higher median rate of telemedicine encounters in the pre–COVID-19 period, but there was no difference in rates by Index of Relative Socioeconomic Disadvantage in subsequent time periods (Figure 4B). There was no evidence of a difference in the proportion of telemedicine encounters related to neighborhood socioeconomic disadvantage across time periods. Children living in Index of Relative Socioeconomic Disadvantage decile 1 had higher median rates of telemedicine (18.4 encounters per 100 persons per month [IQR 14.6-22.3]) than children living in Index of Relative Socioeconomic Disadvantage decile 2 (15.2 encounters per 100 persons per month [IQR 11.4-19.0]).

*Statistical evidence of difference (*P* < .05). ISRD, Index of Relative Socioeconomic Disadvantage.

**Figure 5.**
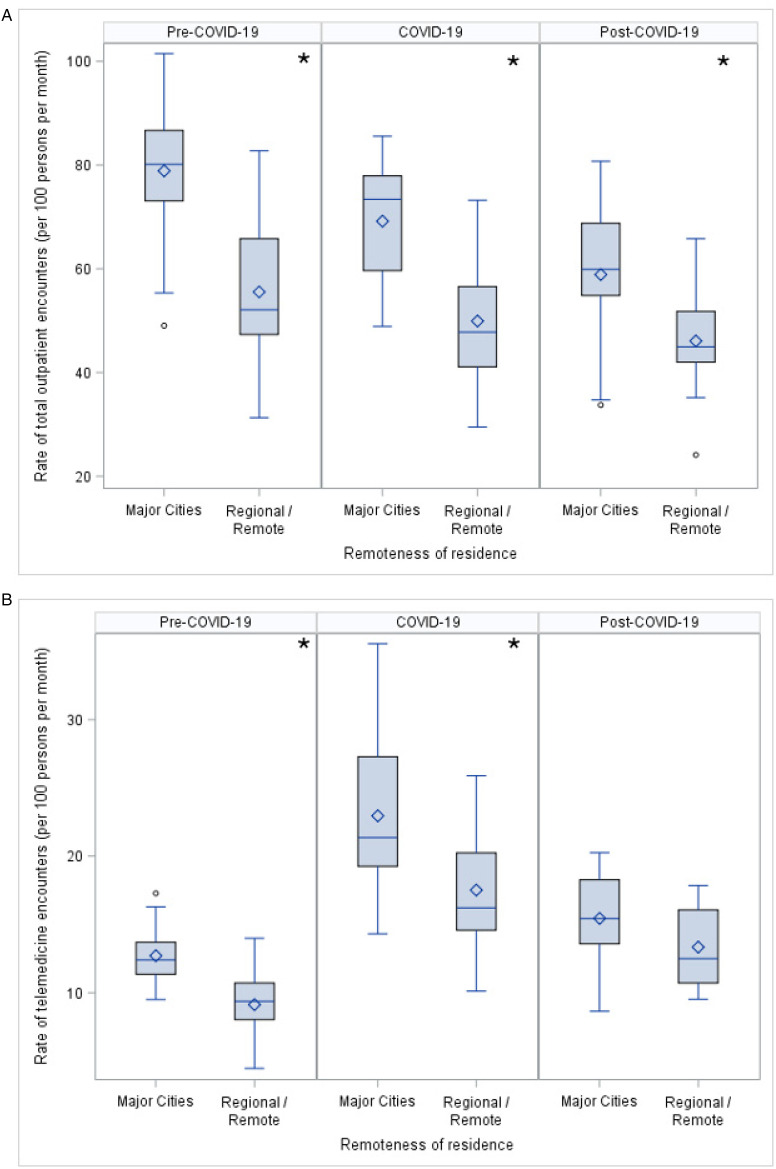
Comparison of (A) rate of outpatient encounters and (B) rate of telemedicine encounters for children with cerebral palsy living in major cities and children with cerebral palsy living in regional and remote areas.

Children with cerebral palsy living in regional and remote areas had lower median rates of total outpatient encounters than those living in major cities during all time periods (Figure 5A). Children living in regional and remote areas also had lower median rates of telemedicine encounters in pre–COVID-19 and COVID-19 periods, but there was no difference post–COVID-19 between groups (Figure 5B). There was no statistical evidence of a difference in the proportion of telemedicine encounters related to geographical remoteness across time periods.

*Statistical evidence of difference (*P* < .05).

## Discussion

The COVID-19 pandemic and associated public health restrictions necessitated a profound change in health service provision including a substantial increase in telemedicine, particularly in high income countries.^23^ Our results illustrate these changes for children with cerebral palsy and suggest, following peaks of telemedicine use during the most severe COVID-19 restrictions in New South Wales, that the use of telemedicine for specialist and allied health outpatient telemedicine consultations for children with cerebral palsy have continued to decline toward prepandemic levels. We found differences in access to telemedicine services for children living in regional and remote areas, with lower rates of total and telemedicine outpatient encounters for these children than those living in major cities before and during COVID-19. This difference persisted for total outpatient encounters post–COVID-19, but the difference narrowed as the rates of telemedicine encounters declined much faster for children living in major cities. We found no differences in access to telemedicine services with children experiencing neighborhood socioeconomic disadvantage. We also found a decline in the frequency of total outpatient encounters for children with cerebral palsy over time, which persisted even when adjusting for the age of the child.

Our findings have some similarities, but also some differences with published telemedicine literature. The peak in telemedicine use at the beginning of the pandemic is similar to that seen in US-based child neurology study,^14^ and consistent with practice internationally.^24,25^ The ongoing decline in telemedicine we have observed is an interesting finding given the political investment in sustaining telemedicine after the pandemic.^26^ Some of this decline may be due to patient and clinician preference for in-person consultations,^27,28^ including for families of children with neurodevelopmental disorders,^15^ reported during the pandemic. Studies have also described challenges that accompanied pandemic telemedicine experiences, including difficulties with managing the technology, difficulties for families to implement and keep children engaged in therapy programs^28,29^ and clinician-reported challenges of physical assessment, and establishing and maintaining rapport,^12,25^ perhaps influenced by (at the time) less familiarity with service delivery model, the requirement for different skills, and the known learning curve.^30^

The differences we have noted in telemedicine use between subspecialties are similar to those in other studies.^14,31^ Some of differences noted are likely to relate to differential reliance on physical examination (eg, allied health, orthopedics), or where an intervention is required (eg, botulinum toxin injections, serial casting). There has been less reporting of more positive experiences of telemedicine in children with cerebral palsy, and our research partners also identified advantages of telemedicine for families, including reducing the burden of in-person attendances and time away from other responsibilities (eg, employment, caring roles), and less likelihood of cancellation related to intercurrent illnesses.

An often-reported benefit of telemedicine is improved patient access to health care by reducing travel.^32,33^ The reduced telemedicine outpatient encounters for children living in regional and remote areas in our study before and during COVID-19 is therefore a key service delivery concern. Potential contributing factors may include known limitations of digital infrastructure in rural areas, and potential differences in digital literacy and access to Internet devices.^34^ Some of these children may also access services local to them and/or outside of Sydney, although availability of specialist pediatric services outside metropolitan areas in Australia is typically limited. These differences warrant further investigation, to ensure that children living in regional and remote areas have access to equivalent quality of care as the whole population.

That we found no evidence of differences in telemedicine use related to socioeconomic disadvantage also requires further investigation. Studies based in other countries have described differences in access to telemedicine related to socioeconomic disadvantage^14^ and race.^35^ It may be that other measures of socioeconomic disadvantage are required to identify where the “digital divide” impacts telemedicine use, or that telephone encounters are less impacted.

The decline in total outpatient encounters for these children over time requires further investigation. Studies have described how outpatient care was postponed for children during the pandemic,^12,36^ and some of this decline (including some children only seen in the pre–COVID-19 time period) may represent delays in the system “catching up.” Of primary consideration is whether these children have unmet medical needs, and whether these have been impacted by less frequent outpatient visits. Alternatively, the reduction may reflect a perceived decreased need for frequent specialist visits and/or better integration with local services. At a service level this reduction may be impacted by known workforce issues in the health system (eg, staff retention) during the time studied (particularly after the pandemic).^37^

Strengths of this study include use of a cerebral palsy register to identify the study population and the time frame enabling a description of telemedicine use before, during, and after the COVID-19 pandemic. Limitations include the restriction to 2 metropolitan specialist children's hospitals. New South Wales has a third pediatric hospital (John Hunter Children's Hospital) that provides a range of specialist outpatient services, and may account for some children seen in the New South Wales / Australian Capital Territory CP Register who are not seen in our cohort. We were not able to measure how organizational factors (eg, availability of telemedicine infrastructure, staffing) changed during the study period, and these are likely to have impacted findings. We have also not collected information on health care provided elsewhere within the public health system, or the primary care and private health systems. The findings of this study relate to the setting: Australia is a high-income country. In other settings, with different health care systems, and in middle- and low-income countries, other factors (eg, licensure, funding) may be relevant to consider.

Further research is needed to better delineate the role(s) of telemedicine for children with cerebral palsy and other neurodevelopmental disorders into the future. First among this is reevaluating the telemedicine preferences of families after the pandemic, as much of the recent research was performed during the extraordinary times during the pandemic.^12^ Research is also required to identify effective digital health technologies that can support the identified challenges of telemedicine (eg, physical examination, developing and maintaining rapport) and to improve the evidence base for interventions provided remotely (telerehabilitation). Studies among children with other chronic health conditions have identified that technology (eg, SMS technology, apps) can promote positive health outcomes when specifically targeted at a component of care.^38,39^ Digital health technology is an acceptable medium for health care for adolescents and young adults,^40^ but must be designed acknowledging concerns around privacy, risks of labeling and identity and accessibility,^41^ and consider equity of data and device access from the outset.^42^ Children and young people living in regional and remote areas must be prioritized in this work.

Clinicians have an important role in promoting telemedicine to improve access to health care. Key activities include ensuring patients, especially those living in regional and remote areas, are informed about the availability of telemedicine, and tailor health care models based on the child's specific needs. Ongoing monitoring of telemedicine effectiveness at a patient level and the development of guidelines that promote best practice in telemedicine health care may help integrate telemedicine into long-term practice.

## Conclusion

Telemedicine has been identified as an important medium to improve access to health care, but our results suggest that in New South Wales, further support will be required to sustain rates of use higher than that seen prepandemic. This support may include reducing barriers to access including broadband Internet, developing best practices for telemedicine, and identifying components of care in children and young people with cerebral palsy that can be addressed through digital health technologies outside of in-person outpatient attendances (eg, coordination of investigations). Further work will be required to help deliver on the promise of telemedicine for children with cerebral palsy.

## Supplemental Material

sj-docx-1-jcn-10.1177_08830738251339960 - Supplemental material for Telemedicine for Children With Cerebral Palsy Before, During, and After the COVID-19 Pandemic: An Australian Cohort Study Supplemental material, sj-docx-1-jcn-10.1177_08830738251339960 for Telemedicine for Children With Cerebral Palsy Before, During, and After the COVID-19 Pandemic: An Australian Cohort Study by Simon P. Paget, Sarah McIntyre, Amy von Huben, Kirsty Stewart, Tracey Williams, Emma Maly, Katrina Ford, Sue Woolfenden and Natasha Nassar in Journal of Child Neurology
